# Financial Burden of Emergency Medicine Residency Applications: Pre-, During, and Post-Pandemic

**DOI:** 10.5811/westjem.46997

**Published:** 2025-09-25

**Authors:** Christopher Zeuthen, Eric Shappell, Daniel J. Egan, Elizabeth Barrall Werley, Alexis Pelletier-Bui, Christopher W. Baugh, Abigail Raynor, Alexis Campbell, Angela P. Mihalic, Andrew D. Luo

**Affiliations:** * Massachusetts General Hospital, Department of Emergency Medicine, Harvard Medical School, Boston, Massachusetts; † Brigham and Women’s Hospital, Department of Emergency Medicine, Harvard Medical School, Boston, Massachusetts; ‡ Penn State Health Milton S. Hershey Medical Center, Department of Emergency Medicine/Penn State College of Medicine, Hershey, Pennsylvania; § Cooper University Hospital/Cooper Medical School of Rowan University, Department of Emergency Medicine, Camden, New Jersey; || University of Texas Southwestern Medical Center, Department of Pediatrics, Dallas, Texas

## Abstract

**Introduction:**

Applying to emergency medicine (EM) residency programs is costly. In the past several years, the EM residency application process has undergone multiple changes in recommendations regarding away rotations and interview format, primarily but not solely driven by COVID-19 restrictions. To date, little is known about the financial impact of these changes on EM applicants. This study assesses recent trends and changes in the costs of the EM residency application.

**Methods:**

We analyzed EM applicant survey data from the Texas STAR (Seeking Transparency in Application to Residency) database from 2019–2024. Application cycles were grouped into three time periods: pre-pandemic (2019–2020), pandemic (2021–2022), and post-pandemic (2023–2024). Applicants’ self-reported data for application fees, away rotation costs, interview costs, and total expenses were analyzed. We conducted Kruskal-Wallis testing to evaluate differences in expense-related variables across the three time periods. We performed post-hoc analysis using the Dunn test if significant differences were detected.

**Results:**

This study included 3,495 EM applicants, which represents 8.4% of the total 41,497 Texas STAR survey respondents from 2019–2024. Average per-applicant total costs were $5,412, $2,076, and $3,156 in the pre-, during-, and post-pandemic application cycles. Self-reported total applicant expenses decreased between the pre- and pandemic period and increased from the pandemic and post-pandemic period (*P* < .01). Applicants had the lowest overall costs in 2021. Away rotation, second look, application costs, interview travel and lodging, and virtual interview costs all reached their lowest levels during the pandemic period (*P* < .01). In the post-pandemic period, travel and lodging costs were higher than pre- and during pandemic levels, while interview costs remained lower due to the continued use of virtual interviews (*P* < .01). Applicants from the Western Region of the US saw the highest total costs compared to the Northeast, which saw the lowest.

**Conclusion:**

The total expenses reported by medical students applying to EM residency programs were significantly reduced during the pandemic, compared to other years. Some expenses, notably away rotation and second look and application costs, have risen post-pandemic. To help reduce the financial burden of the EM residency process, the continued use of virtual interviews is an opportunity for cost savings.

## INTRODUCTION

Applying to residency programs is a costly endeavor. Historically, EM applicants have numerous application-related expenses including interview travel and away rotations to obtain a Standardized Letter of Evaluation (SLOE), which is an important component of the application process.^1^–^3^ Prior studies showed that the average pre-pandemic cost exceeded $8,000 per applicant in EM, over half of which stemmed from applications costs and interview-related travel expenses.^4^,^5^ The COVID-19 pandemic brought significant changes to graduate medical education recruitment, evaluation, and selection of applicants. Away rotations were temporarily suspended, and applicant interviews shifted from the traditional in-person format to virtual platforms.^6^–^9^ Although away rotations have largely resumed since 2023, recommendations such as virtual interviews have remained.

Before the 2020–2021 application cycle, advising for EM recommended that most applicants complete two core EM rotations, and most interviews were conducted in person.^10^ In 2020–2021, due to widespread COVID-19 restrictions and national recommendations, EM programs supported applicants completing only one home rotation, a policy shift seen across many specialties.^11^ Since the 2021–2022 cycle, guidance regarding away rotations has returned to pre-pandemic norms while maintaining virtual interviews.^3^

To help offset applicants’ financial burden, several evolving measures have been taken on the national or institutional level to help applicants navigate application costs. For example, the Association of American Medical Colleges (AAMC) revised the fee structure for the 2025–2026 application cycle. In addition, some specialties, including obstetrics/gynecology and EM, have transitioned from the Electronic Residency Application Service to Residency Centralized Application Service (ResidencyCAS), a centralized application system that may reduce applicant costs by up to 20%..^12^,^13^ Organizations such as the Council of Residency Directors in Emergency Medicine and the Emergency Medicine Residents’ Association have incorporated financial planning tips specific to EM into student advising guides.^14^ Lastly, there is growing availability of diversity, equity, and inclusion scholarships for applicants participating in away rotations.^15^

Over the course of these changes, little has been reported about the collective impact on the financial burden incurred by EM residency applicants. In addition, no reports examine how the costs for applicants vary across geographic regions or application years. Prior reports of EM residency application costs predate COVID-19-related changes, which underscores the need for updated research on the current financial landscape for EM. In this study we aimed to assess the changes in EM residency application costs over the pre-, during, and post-pandemic periods. Insights from our study may help stakeholders in the EM residency application process determine ways to better mitigate costs to applicants in the future, creating a more equitable residency application process.

**Population Health Research Capsule**

What do we already know about this issue?

*Residency application costs are high and can vary across years, regions, and interview format*.

What was the research question?

*How did average emergency medicine (EM) residency application costs change across the COVID-19 pandemic?*

What was the major finding of the study?

*Mean total costs: $5,412 (pre); $2,076 (during); and $3,156 (post-pandemic); P < .001*.

How does this improve population health?

*Sustaining virtual interviews may reduce cost burden and promote equity in EM residency access*.

## METHODS

We conducted a retrospective, cross-sectional analysis of the Texas Seeking Transparency in Application to Residency (STAR) database. The Texas STAR database, managed by the University of Texas Southwestern Medical Center in Dallas, TX, is a nationwide resource that compiles self-reported data from medical students at participating schools regarding their residency applications.^16^ The database represents six years of data from 155 medical schools. Data include application characteristics, expense variables, geographic region variation in costs, and other metrics.

### Study Population

We evaluated survey data for EM residency applicants from the 2019–2024 application cycles. In this study, “cycle” refers to the year applicants matched and were slated to start residency training (eg, the 2024 cycle refers to the 2023–2024 application cycle, during which applicants applied in 2023 and then matched and entered residency programs in 2024). Our analysis included both US allopathic (MD) and osteopathic (DO) senior medical students. Responses from international medical graduates (IMG), previous graduates, and reapplicants are not collected in the Texas STAR database and, therefore, were not included in this study. Survey applicant demographic information was broken down by geographic region using the AAMC Group on Student Affairs classifications: Central; Northeastern; Southern; and Western.^17^

### Variables

We collected demographic data for sex and under-represented in medicine status (URM) and academic metrics, such as the US Medical Licensing Examination (USMLE) Step 1 pass-fail, USMLE Step 2 Clinical Knowledge score, honor society membership in Alpha Omega Alpha or Gold Humanism (Table 1). Additionally, we collected application metrics, including the number of programs applied to, interview offers, and interviews attended. Lastly, Texas STAR collected several expense-related variables: travel cost; application fees; virtual interview cost; away rotation and second-look cost (collected as a single variable); and total cost; reported in US dollars within a range of values (eg, $500–$1,000).

### Statistical Analysis

We categorized the 2019–2024 application cycles into three periods: pre-pandemic (2019–2020); pandemic (2021–2022); and post-pandemic (2023–2024). This categorization was chosen to reflect changes driven by the pandemic and changes in program recruitment practices and recommendations during these periods.

To compare the range of costs reported within expense-related variables, we calculated a weighted mean and standard deviation for each period’s expense variables, and the total reported costs in each geographic region, enabling comparisons for each group. These weighted means and SDs are presented in the tables and results. All values are reported in the format of weighted mean ± SD in our methods section. For a weighted average calculation, we assigned weights (number of applicants) to each value (midpoint of the range) in a set, after which they were averaged together and compared between years (represented as the weighted mean difference). Weighted mean = [sum of (value × weight)] / (sum of weights). We used a Shapiro-Wilk test to assess the normality of the data distribution. As the expense-related variables were not normally distributed, we applied non-parametric methods. A Kruskal-Wallis test was conducted to evaluate differences in expense-related variables across the three time periods. We performed post-hoc analysis using the Dunn test if significant differences were detected.

Lastly, we recognized inflationary changes throughout the three periods. To perform a sensitivity analysis, we adjusted the expense variables in each year to the 2024 real costs by applying the Consumer Price Index (CPI) as the inflation adjustment factor. Specifically, expenses from previous years were multiplied by the ratio of the CPI in 2024 to the CPI of the respective year, standardizing all costs to reflect their real value in 2024 US dollars (Supplemental Table 1a and 1b). We used R programming software v2023.12.0, (The R Foundation for Statistical Computing, Vienna, Austria) for all statistical analysis. The data used in this study did not meet the criteria for human subject research by the Mass General Brigham Institutional Review Board, and the study was deemed exempt.

## RESULTS

From 2019 to 2024, 41,497 students completed the Texas STAR survey, including 3,495 EM applicants (8.4%). United States MD seniors comprised nearly 100% of respondents pre-pandemic and approximately 80% post-pandemic, with a corresponding increase in DO respondents. Regional representation remained stable across application cycles, with applicants from the Western US representing the smallest proportion of respondents (Table 1).

**Figure 1:**
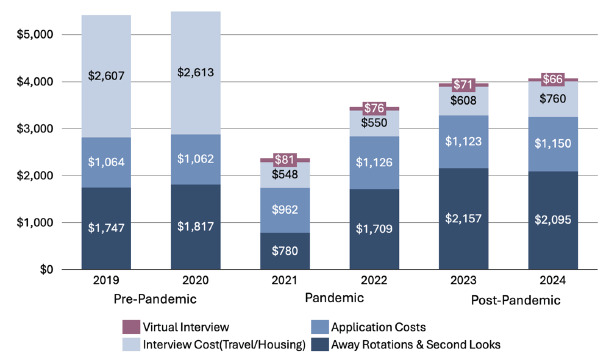
Emergency medicine residency applicants’ average cost, by category (in United States dollars).

**Figure 2:**
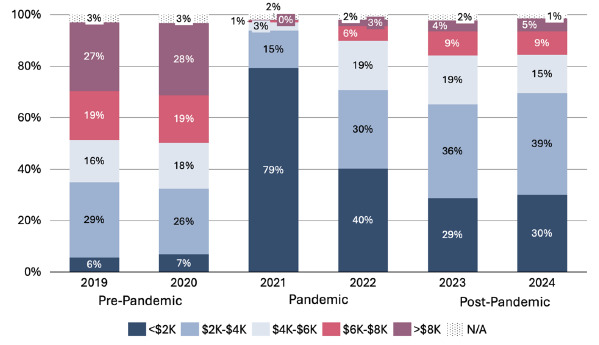
Distribution of emergency medicine residency applicants’ self-reported total application costs, in $2,000 increments, by application year (%). *N/A*, no answer.

Average total application costs were $5,412 ± $3,481 pre-pandemic, $2,076 ± $1,735 during the pandemic, and $3,156 ± $2,549 post-pandemic, representing a 62% drop during the pandemic and a 42% reduction from pre-pandemic levels (Table 2a, Figure 1). The greatest savings came from costs related to interview travel and lodging, which declined from $2,610 ± $2,276 pre-pandemic to $549 ± $353 during the pandemic. In 2021, at the height of COVID-19, 79% of applicants reported total spending of < $2,000. with 1% reporting spending > $6000. By 2024, 14% of applicants reported spending > $6,000, and 30% reported total costs of < $2000 (Figure 2).

Away-rotation and second-look costs decreased from $1,787 ± $1,498 pre-pandemic to $1,388 ± $1,384 during the pandemic but rose post-pandemic to $2,123 ± $1,828, surpassing pre-pandemic levels. In comparison, application costs followed a similar trend with post-pandemic costs reaching a high of $1,138 ± $647 and a low of $1,041 ± $682 during the pandemic (Table 2a–b, Figure 1).

Pre-pandemic applicants reported spending on average $2,610 ± $2,276 on interview travel and lodging costs, peaking in 2020 at $2,613 ± $2,303. During the pandemic, interview costs declined, with an average cost of $549 ± $353, with a low of $548 ± $427 in 2021. Post-pandemic interview travel and lodging costs rose to $700 ± $636, which remain below pre-pandemic levels. With a shift to primarily virtual interviews during and after the pandemic, applicant costs across these two periods were $79 ± $51 and $69 ± $42, respectively (Table 2a–b, Figure 1).

The Kruskal-Wallis test found statistical significance for all cost variables (*P* < .001). Post-hoc analysis identified significant differences between pre-, during, and post-pandemic periods for total application costs, away-rotation and second-look costs, virtual interviews, and interview travel and lodging costs. The one outlier was application costs. The post-hoc analysis did not reveal statistical significance between pre- and pandemic period application costs (*P* = .23), but it did for pre- vs post-pandemic and the pandemic vs post-pandemic periods (P < .001).

Applicants from the Western region reported the highest total application costs across all periods: $7,744 ± $4,535 pre-; $3,077 ± $2,572 during; and $4,617 ± $3,459 post-pandemic. In comparison, applicants from the Northeast maintained the lowest average total costs during these periods, with a low at $1,781 ± $1,394 during the pandemic. Total costs declined during the pandemic across all regions and rose post-pandemic but remained below pre-pandemic levels. The Kruskal-Wallis test confirmed significant differences in total costs across the three periods within each region (*P* < .001) and Dunn post-hoc analysis identified significant pairwise differences between each region for each pandemic period (*P* < .001) (Table 3). Sensitivity analysis, grouped by pandemic period and adjusted to 2024 dollars to account for inflation, showed consistent statistical significance across all expense variables (Supplemental Table 1a–b).

## DISCUSSION

In this study we evaluated recent cost trends in the EM residency application and interview process, considering COVID-19 restrictions and policy changes. This is the first study to assess EM residency application expenditures across various cost categories, offering a national analysis of how COVID-19 policies affected applicants’ expenses.

Away rotations and second looks remain costly but represent impactful elements according to program directors and, therefore, represent necessary expenses.^2^ Initiatives like broader visiting student scholarships and subsidized housing have helped reduce these costs, but they remain limited.^15^ Continued advising regarding judicious use of away rotations is helpful. During the 2021 and 2022 application cycles, costs for away rotations and second looks significantly decreased, likely due to pandemic-related restrictions. The future impact of post-pandemic changes on these costs is uncertain. Notably, the Texas STAR database combines away rotations and second looks as one cost. The former occurs early and is an essential component, while the latter occurs later in the application season and is optional, yet both incur travel and lodging expenses.

Virtual interviews have helped minimize costs for applicants, as virtual interview equipment is inexpensive and reusable throughout the interview season. The shift to virtual interviews has increased accessibility, allowing applicants to attend more interviews, and enabled programs to interview more candidates, as shown in our analysis (Table 1). Many program directors note that virtual interviews sufficiently validate their perceptions of applicants.^18^–^20^ While further research is necessary to assess the effectiveness of virtual interviews in evaluating candidates and determining programs’ rank order lists, EM continues to support virtual interviews for reasons including applicant equity.^21^

Given the high costs of away rotations and second looks identified in our study, applicants could allocate financial savings from virtual interviews to participate in second-look days. Unlike pre-pandemic applicants who interviewed in person, post-pandemic applicants may rely more on second-look experiences to assess programs, a trend noted in other specialties.^22^,^23^ Additionally, with the potential increase in second-look participation and the return of away rotations, applicants face rising travel and accommodation costs in the post-pandemic era.^24^ The future of virtual interviews remains uncertain, but their recent implementation has noticeably impacted costs.^25^

Similar to the findings in our dataset (Table 1), applications per applicant have decreased in the past several years from their peak in 2022, suggesting that the per-application cost has steadily risen.^26^ While ERAS fees per application increased incrementally across tiers under the previous structure (eg, $99 for the first 10 programs, then $26 per program beyond 30 applications in the 2021–2022 cycle), the AAMC implemented a simplified fee model beginning with the 2025 application cycle, setting costs at $11 per program for the first 30 programs and $30 thereafter. These structural changes may have influenced applicant behavior, although causality cannot be determined from the available data.^12^ Our findings align, at least in part, with national AAMC EM data, suggesting that our findings may be reflective of national trends; however, further extrapolation to the broader EM application pool would be required to confirm generalizability. Another factor contributing to the rising costs demonstrated in our dataset for the post-pandemic period is the proportion of DO applicants, which rose from near 0% in the pre-pandemic period to approximately 20% post-pandemic (Table 1). With osteopathic medical schools more geographically dispersed and with fewer residency program affiliations DO applicants may incur higher costs for traveling to away rotations and interviews.^27^ This evolving applicant pool may partially contribute to cost differences observed over time.

Several initiatives aim to reduce applicant costs, including the AAMC’s revised ERAS fee structure for the 2025 application cycle and the planned transition to ResidencyCAS by some programs in 2025–2026, which is projected to cut average application fees by up to 20%.^12^–^13^ However, these changes address only one part of the cost burden—primarily application submission fees. More expensive components, such as travel and lodging for away rotations, in-person interviews, and second looks, remain largely unchanged. While national guidelines recommend continuing virtual interviews to reduce costs, programs retain flexibility in their interview format. Given the significant financial savings associated with virtual interviews, they should be considered a key strategy to reduce applicant burden. As programs increasingly explore hybrid or in-person approaches, continued monitoring of total application and interview costs will be important. In addition, since application costs are self-reported, they may include broader expenses such as Visiting Student Learning Opportunities (VSLO) fees or ancillary services.

Applicants across all geographic regions experienced cost savings during the pandemic as away rotations and second looks were paused and virtual interviews were introduced. Despite the return of away rotations and some in-person second looks, applicants across all regions reported lower total costs post-pandemic, largely driven by reduced interview costs likely due to continuing virtual interviews. Western region applicants have particularly benefited from the transition to virtual interviews, as pre-pandemic, they demonstrated the highest travel and lodging expenses. However, Western region applicants still report the highest overall costs compared to other regions, especially those in the Northeast.

Regional cost differences are most likely driven by away-rotation and second-look attendance collectively. With many EM programs concentrated on the East Coast, Western region applicants face heightened travel and accommodation costs to attend away rotations and second looks.^28^ In comparison, Northeastern applicants can more readily commute regionally for away rotations and second looks. Moreover, the 2022–2023 ERAS applicant survey shows that 69% of EM applicants state that geographic location was the most important factor in where they applied.^29^ Finally, EM organizations and residency programs could continue to seek other ways to reduce applicant costs, such as grants, and to emphasize that second looks are non-evaluative and optional, to minimize unnecessary expenses.

The reported costs in our analysis need to be considered in the context of inflation. Over the application years 2019–2024 examined in our study, the CPI ranged from 255.7 to 314.4, rising year over year to a high in 2024.^30^ Notably, we performed a sensitivity analysis to account for these changes, which yielded results consistent with our unadjusted analysis, highlighting that inflation did not substantially alter our observed trends or statistical significance across the examined periods.

Lastly, with the upcoming transition of EM to the ResidencyCAS platform, there will be a structural shift in the application process. This change is imminent and raises important questions about future cost implications However, it is important to note that ResidencyCAS addresses only one component of the overall cost burden—primarily application submission fees. Our study highlights that the most significant cost drivers for EM applicants have historically been away rotations and travel, which are not directly affected by the choice of application platform. Therefore, we hope that our findings will help inform future advising, equity discussions, and cost-containment strategies even as the logistics of application submission evolve.

## LIMITATIONS

There are several limitations to this study. While the Texas STAR survey is a national database, survey respondents were primarily US MD seniors, with a smaller proportion of US DO seniors. It does not represent all US medical schools nor does it entail a proportional distribution of applicants across all geographic regions. Further, the study did not include responses from IMGs or those applicants who may have previously graduated from medical school. Osteopathic students and IMGs represent a substantial percentage of applicants to EM residency positions in a shifting applicant pool.^25^ In addition, with fewer home programs these applicants will require two away rotations to obtain the recommended two SLOEs. Notably, DO and IMG applicants often perform more away rotations due to fewer home EM programs, which may result in higher unmeasured costs. Thus, our calculated averages may underestimate true costs for the broader applicant population.

In addition, because the Texas STAR survey is anonymous and voluntary, it is susceptible to response bias. Respondents who choose to complete the survey may differ systematically from non-respondents in ways that affect cost reporting. Furthermore, because the survey is completed several months after expenses are incurred, the data are subject to recall bias. Another limitation is that the Texas STAR survey combines away rotation and second-look events into one cost variable when these are two separate events during an applicant’s cycle. Given the combined variable, it is challenging to determine which of these is the primary driver of costs. Additionally, the components contributing to “virtual interview costs” are not well-defined, and we were unable to verify what applicants chose to include in this category.

While in-person costs (eg, travel, lodging) are more straightforward, virtual costs may have included equipment, software, or internet upgrades. Finally, the Texas STAR survey does not account for instances by which applicants may have reduced their total expenditures, such as visiting student scholarships, housing discounts, or sublet/house swap opportunities, which may alleviate some of the costs reported by applicants.

## CONCLUSION

Emergency medicine applicant costs were lowest during the pandemic, primarily due to travel restrictions and the shift to virtual interviews. Post-pandemic costs rose but remained below pre-pandemic levels overall, with increases in application, away rotation, and second-look expenses. The continuation of virtual interviews appears to be a key strategy for reducing applicant financial burden.

## Supplementary Information



## Figures and Tables

**Table 1:** Demographics and characteristics of respondents to the Texas Seeking Transparency in Application to Residency survey: 2019–2024.

	Pre-Pandemic		Pandemic		Post-Pandemic	
	2019	2020	2021	2022	2023	2024
Texas STAR National Dataset						
Total number of students who received survey	15,404	15,783	17,179	18,661	20,386	22,640
Survey respondents	5,864	7,418	6,912	7,073	6,962	5,901
Response rate	38%	47%	40%	38%	34%	26%
Texas STAR - Emergency Medicine Respondents						
Total EM Respondents	504	652	687	637	478	489
Male (%)^1^	N/A	N/A	N/A	253 (39.7%)	166 (34.7%)	177 (36.2%)
Female (%)^1^	N/A	N/A	N/A	253 (39.7%)	189 (39.5%)	216 (44.2%)
Unknown sex / Prefer not to Say (%)^1^	504 (100%)	652 (100%)	687 (100%)	130 (20.4%)	121 (25.3%)	96 (19.6%)
Under-represented in Medicine %^1^	N/A	N/A	N/A	87 (13.7%)	64 (13.4%)	46 (8.6%)
US MD senior (%)	504 (100%)	642 (98.5%)	635 (92.4%)	569 (89.3%)	393 (82.2%)	384 (78.5%)
US DO senior (%)	N/A	10 (1.5%)	52 (7.6%)	68 (10.7%)	85 (17.8%)	105 (21.5%)
Number of programs applied to (SD)	49.4 (±22.5)	50.2 (±23.2)	48.1 (±22.9)	52.7 (±22.2)	48.7 (±20.9)	41.0 (±20.1)
Number of interview offers (SD)	20.1 (±16.3)	20.0 (±9.8)	18.4 (±9.6)	21.2 (±10.5)	26.0 (±11.9)	21.3 (±9.7)
Numbers of interviews attended (SD)	12.9 (±4.8)	13.2 (±4.2)	13 (±5.5)	15.0 (±5.6)	16.5 (±6.4)	15.7 (±6.0)
USMLE Step 1 pass rate, 1^st^ attempt (%)	494 (98%)	641 (98.3%)	659 (95.9%)	616 (96.7%)	446 (93.3%)	489 (91.1%)
Average Step 2 CK Score (SD)	248.3 (±12.5)	248.6 (±12.8)	249.5 (±12.8)	248.4 (±12.6)	247.8 (±13.1)	249.1 (±12.4)
AOA membership (%)	79 (15.7%)	97 (14.9%)	105 (15.3%)	87 (13.7%)	96 (20.1%)	111 (20.7%)
Gold Humanism Honor Society membership (%)	76 (15.1%)	102 (15.6%)	129 (18.8%)	116 (18.2%)	85 (17.8%)	69 (12.8%)
Couples matching (%)	39 (7.7%)	54 (8.3%)	57 (8.3%)	40 (6.3%)	39 (8.2%)	45 (8.4%)
Peer-reviewed publications (SD)	1.1 (±1.8)	1.3 (±2.0)	1.7 (±2.4)	1.7 (±2.3)	1.7 (±2.4)	1.5 (±2.4)
Research experiences (SD)	2.9 (±2.2)	3.0 (±2.0)	3.2 (±2.3)	3.2 (±2.1)	3.2 (±2.5)	2.9 (±2.5)
Clerkship honors (SD)	3.2 (±2.4)	3.1 (±2.4)	3.1 (±2.3)	3.0 (±2.4)	2.9 (±2.5)	3.4 (±2.5)
Geographic Region						
Central	123 (24%)	167 (26%)	155 (23%)	148 (23%)	126 (26%)	157 (32%)
Northeast	122 (24%)	174 (27%)	192 (28%)	164 (26%)	106 (22%)	97 (20%)
South	199 (39%)	222 (34%)	259 (38%)	208 (33%)	173 (36%)	171 (35%)
West	60 (12%)	89 (14%)	81 (12%)	117 (18%)	73 (15%)	64 (13%)

1 Sex and under-represented in medicine demographic information was not collected on the Texas STAR database until 2022.

*AOA*, Alpha Omega Alpha*; EM*, emergency medicine; *MD*, doctor of medicine; *DO*, doctor of osteopathic medicine; *STAR*, Seeking Transparency in Application to Residency; *USMLE*, US Medical Licensing Exam; *CK*, clinical knowledge.

**Table 2a:** Emergency medicine residency applicants’ yearly average cost with standard deviation, by category ($US).

Expense variable	Pre-Pandemic		Pandemic		Post-Pandemic	
	2019	2020	2021	2022	2023	2024
Away rotation and second looks	$1,747 ± 1,380	$1,817 ± 1,582	$780 ± 650	$1,709 ± 1,552	$2,157 ± 1,736	$2,095 ± 1,904
Application costs	$1,064 ± 706	$1,062 ± 654	$962 ± 671	$1,126 ± 684	$1,123 ± 696	$1,150 ± 601
Interview (travel and lodging)	$2,607 ± 2,243	$2,613 ± 2,303	$548 ± 427	$550 ± 268	$608 ± 514	$760 ± 698
Virtual interview	N/A	N/A	$81 ± 55	$76 ± 47	$71 ± 44	$66 ± 40
Total application costs	$5,351 ± 3,439	$5,460 ± 3,515	$1,402 ± 1,074	$2,803 ± 2,000	$3,501 ± 2,380	$3,530 ± 2,691

**Table 2b:** Emergency medicine residency applicants’ average cost with standard deviation, by cost category ($US) and pandemic period.

Expense variable	Pre-Pandemic	Pandemic	Post-Pandemic	*P*-value (Pre- vs During)	*P*-value (During vs Post)	*P*-value (Pre- vs Post)
Away rotation and second looks	$1,787 ± 1,498	$1,388 ± 1,384	$2,123 ± 1,828	< .001	< .001	< .001
Application costs	$1,063 ± 677	$1,041 ± 682	$1,138 ± 647	.23	< .001	< .001
Interview (Travel and lodging)	$2,610 ± 2,276	$549 ± 353	$700 ± 636	< .001	< .001	< .001
Virtual interview	N/A	$79 ± 51	$69 ± 42	N/A	< .001	N/A
Total application costs	$5,412 ± 3,481	$2,076 ± 1,735	$3,516 ± 2,549	< .001	< .001	< .001

**Table 3:** Emergency medicine residency applicants’ average cost with standard deviation, by category ($USD*) organized by pre-pandemic (2019–2020), pandemic (2021–2022), and post-pandemic (2023–2024), and by AAMC Student Affairs Region.

Geographical region	Pre-Pandemic	Pandemic	Post-Pandemic	*P*-value (Pre- vs During)	*P*-value (During vs Post-)	*P*-value (Pre- vs Post-)
Central	$5,832 ± 3,862	$2,101 ± 1,800	$3,166 ± 2,177	< .001	< .001	< .001
Northeastern	$5,084 ± 3,379	$1,781 ± 1,394	$2,898 ± 2,087	< .001	< .001	<.001
Southern	$6,836 ± 4,390	$2,248 ± 1,806	$3,525 ± 2,472	< .001	< .001	< .001
Western	$7,744 ± 4,535	$3,077 ± 2,572	$4,617 ± 3,459	< .001	< .001	< .001

* *USD*, United States dollars; *AAMC*, Association of American Medical Colleges.
