# Supplementary material for: Financial Burden of Emergency Medicine Residency Applications: Pre-, During, and Post-Pandemic
Source: West J Emerg Med. 2025 Sep 25;26(5):1154–61. doi: 10.5811/westjem.46997 (PMC12591620; doi:10.5811/westjem.46997)
Supplement: Supplementary file 1 [file wjem-26-1154-s001.docx]

**Supplemental Table 1: (a)** Emergency Medicine Residency Applicants’ Yearly Average Cost, by Category ($USD) with inflation adjustment **(d)** Average Cost, by Category ($USD) with inflation adjustment grouped by pandemic period

**Supplemental Table 1a:**

|  | Pre-Pandemic | | Pandemic | | Post-Pandemic | |
| --- | --- | --- | --- | --- | --- | --- |
| Expense Variable | 2019 | 2020 | 2021 | 2022 | 2023 | 2024 |
| Away Rotation & Second Looks | $2,085 ± 1647 | $2,169 ± 1889 | $848 ± 707 | $1,860 ± 1,689 | $2,157 ± 1,736 | $2,095 ± 1,904 |
| Application Costs | $1,269 ± 842 | $1,268 ± 781 | $1,047 ± 730 | $1,225 ± 745 | $1,123 ± 696 | $1,150 ± 601 |
| Interview (Travel & Housing) | $3,111 ± 2677 | $3,119 ± 2748 | $596 ± 465 | $598 ± 291 | $608 ± 514 | $760 ± 698 |
| Virtual Interview | N/A | N/A | $88 ± 60 | 83 ± 51 | $71 ± 44 | $66 ± 40 |
| Total Application Costs | $6,387 ± 4104 | $6,516 ± 4195 | $1,526 ± 1,169 | $3,050 ± 2,176 | $3,501 ± 2,380 | $3,530 ± 2,691 |

**Supplemental Table 1b:**

| Expense Variable | Pre-Pandemic | Pandemic | Post-Pandemic | P-value (Pre- vs. During-) | P-value (During vs. Post) | P-value (Pre- vs. Post) |
| --- | --- | --- | --- | --- | --- | --- |
| Away Rotation & Second Looks | $2,133 ± 1,788 | $1,510 ± 1,506 | $2,123 ± 1,828 | <0.001 | <0.001 | <0.001 |
| Application Costs | $1,268 ± 808 | $1,133 ± 742 | $1,138 ± 647 | 0.68* | <0.001 | <0.01 |
| Interview (Travel & Housing) | $3,116 ± 2716 | $597 ± 384 | $700 ± 636 | <0.001 | <0.001 | <0.001 |
| Virtual Interview | N/A | $85 ± 56 | $69 ± 42 | N/A | <0.001 | N/A |
| Total Application Costs | $6,459 ± 4,154 | $2,259 ± 1,888 | $3,516 ± 2,549 | <0.001 | <0.001 | <0.001 |
